# Area deprivation and the food environment over time: A repeated cross-sectional study on takeaway outlet density and supermarket presence in Norfolk, UK, 1990–2008

**DOI:** 10.1016/j.healthplace.2015.02.012

**Published:** 2015-05

**Authors:** Eva R. Maguire, Thomas Burgoine, Pablo Monsivais

**Affiliations:** UKCRC Centre for Diet and Activity Research (CEDAR), MRC Epidemiology Unit, University of Cambridge School of Clinical Medicine, Institute of Metabolic Science, Box 285, Cambridge Biomedical Campus, Cambridge CB2 0QQ, UK

**Keywords:** Food environment, Takeaway food outlet, Supermarket, Socioeconomic status, Inequalities

## Abstract

Socioeconomic disparities in the food environment are known to exist but with little understanding of change over time. This study investigated the density of takeaway food outlets and presence of supermarkets in Norfolk, UK between 1990 and 2008. Data on food retail outlet locations were collected from telephone directories and aggregated within electoral wards. Supermarket presence was not associated with area deprivation over time. Takeaway food outlet density increased overall, and was significantly higher in more deprived areas at all time points; furthermore, socioeconomic disparities in takeaway food outlet density increased across the study period. These findings add to existing evidence and help assess the need for environmental interventions to reduce disparities in the prevalence of unhealthy food outlets.

## Highlights

• We examined disparities in the retail food environment over 18 years in Norfolk, UK
• Supermarket presence was not associated with area-level deprivation
• Takeaway food outlet density was significantly greater in more deprived areas
• Socioeconomic inequalities in takeaway food outlet density increased over time

## 1 Introduction

Social ecological models emphasise the multiple spheres of influence on health behaviours including the physical neighbourhood environment (Sallis et al., 2008; Swinburn et al., 1999). In relation to dietary behaviours, the local food retail environment, or ‘foodscape’ (Lake et al., 2010), is considered unsupportive of healthful food choices when energy-dense foods of low nutritional value are readily available and when there are few opportunities to purchase healthier foods (Swinburn et al., 2013). Takeaway food (׳fast food׳) outlets primarily offer ready-to-eat, energy-dense foods, which are associated with higher total energy and fat intakes (Lachat et al., 2012). Frequent consumption of takeaway food has been associated with excess weight gain over time (Rosenheck, 2008; Prentice and Jebb, 2003). Given the substantial growth in the number of takeaway food outlets in recent years in the UK (The Strategy Unit, 2008), one possibility is that increasing exposure to these food outlets is associated with excess consumption of takeaway foods and excess body weight (Burgoine et al., 2014). In contrast, access to supermarkets might be important in enabling healthful food purchasing as they generally offer a wide range of products at a number of price points (Morland et al., 2006; Burns and Inglis, 2007). Although research findings remain equivocal, neighbourhood access to supermarkets has been associated with lower obesity rates in the international literature (Lovasi et al., 2009; Feng et al., 2010), and supermarkets account for the majority of food bought to consume at home in the UK (Defra, 2014). Like the takeaway sector, supermarkets have changed in recent decades in the UK, with large supermarkets increasingly built on the edge of urban areas during the 1990s (White, 2007). The large range of products sold and low prices in these out-of-town outlets challenged smaller town and city centre food retailing (Wrigley, 2002). The period of the 1990s and 2000s is therefore one in which shifts in the foodscape may be expected.

Being overweight and the consumption of unhealthy diets are found disproportionately among those with low socioeconomic status (SES) and living in deprived areas (Ellaway et al., 1997; Van Lenthe and Mackenbach, 2002; Dowler, 2001). One hypothesis is that less healthy foodscapes in deprived neighbourhoods could be contributing to established and widening socioeconomic gradients in diet and health. Thus, whether exposure to certain food outlets varies by area deprivation is of public health concern. Inequalities in takeaway food outlet exposure have been identified in the United Kingdom (Macdonald et al., 2007; Cummins et al., 2005), New Zealand (Pearce et al., 2007, 2008), Australia (Reidpath et al., 2002), Canada (Smoyer-Tomic et al., 2008), and the United States (Morland et al., 2002; Richardson et al., 2012; Powell et al., 2007; Block et al., 2004). There is general consensus in the review literature that there is poorer supermarket availability in more deprived areas in the United States (Black et al., 2014; Larson et al., 2009), while a small number of studies from Canada have suggested restricted supermarket access for low-income groups (Bertrand et al., 2008; Larsen and Gilliland, 2008). Access to all outlet types, including supermarkets, was found to be greater in more deprived areas in New Zealand (Pearce et al., 2008), while in one UK study no disparities in supermarket access were identified (White et al., 2004). With two known exceptions (Pearce and Day, 2010; Richardson et al., 2014) the existing literature has not explored change in these disparities over time. Yet with increasing focus on the role of the food environment in shaping food choices it is important to more fully characterise the association between food access and deprivation in the UK.

The aims of this study were twofold: to assess the area-level density of takeaway food outlets and presence of supermarkets with respect to deprivation over time in one area of the UK, and to examine deprivation-specific food environment stability in the same neighbourhoods over time.

## 2 Methods

The study area was Norfolk, a large county situated in the East of England with a resident population in 2001 of 796,728 (Office for National Statistics, 2001). Similar to other English counties, Norfolk is comprised of both rural and urban areas, with 32% of electoral wards containing more than 10,000 residents (Office for National Statistics, 2004). Norfolk contains only one major city, Norwich, which had a resident population of 122,400 in 2001 (Office for National Statistics, 2001).

### 2.1 Data collection

We collected food outlet data for Norfolk from Yellow Pages telephone directories archived at the Millennium Library in Norwich. We systematically recorded the names and addresses of all food-related outlets across six available time points (1990, 1992, 1996, 2000, 2003 and 2008). Complete addresses (83%) were subsequently geocoded using an address lookup table for Norfolk, provided by Ordnance Survey. Where the Yellow Pages record provided no street number or building name (12%), we geocoded the address as number ‘1’ (e.g. ‘1 High Street’ if listed as ‘High Street’). A sensitivity analysis suggested that the error arising from misclassifying outlet addresses to an incorrect deprivation tertile was likely to be minimal (Supplementary Appendix 1). Where an address was not present in the lookup file (5%), we used the internet to find a postcode for the premises, which was then geocoded using GeoConvert (http://geoconvert.mimas.ac.uk/). UK postcodes allow for relatively precise geocoding, with each postcode containing on average only 15 addresses (Smith et al., 2013).

### 2.2 Food outlet classification

We reclassified outlets from the existing Yellow Pages classifications. We operationalised ‘Supermarket’ to include national retailers with a substantial share of the food and non-alcoholic drinks market, and excluded specialist frozen food stores offering restricted grocery lines not comparable with the larger retailers. Eight retailers were included in total. The four largest national retailers were listed as Tesco, Sainsbury׳s, ASDA and Morrisons by the Department for Environment, Food and Rural Affairs (Defra), which in 2008 had a combined market share of 75.6%. Three further retailers – Waitrose, Somerfield (formerly Gateway), and the Co-operative, also known regionally as Anglia Regional Co-op – had a smaller but sizeable combined market share of 11.9% (Defra, 2008). We also included Safeway, a major retailer in the early 1990s (Burgoine et al., 2009) and the early 2000s (Clarke et al., 2002). While we did not classify supermarkets based on market share from previous years, the supermarket sector in the UK was fairly stable over the time period of interest. The four largest retailers in 2008 were, along with Safeway, the major stores for domestic groceries before and into our study period (Clarke et al., 2002).

Our ‘Takeaway’ food outlet category corresponded with UK planning terms, defined under the Town and Country Planning Use Classes Order 1987 (as amended). A Class A5 hot food takeaway is defined as an outlet whose primary business is ‘the sale of hot food for consumption off the premises’ (Planning Portal, 2014). Characteristics of these takeaway food outlets include: hot food is ordered and paid for at the till, limited space for sitting in, and no waiter service (Lake et al., 2010). As such, examples of takeaway food outlets included fried chicken, fish and chip, pizza or kebab shops, Indian and Chinese takeaways (London Borough of Barking and Dagenham, 2010), and did not include cafes, full service restaurants, drinking establishments and shops. Major takeaway food franchises (McDonalds, Burger King, KFC, Wimpy) were classified in some editions of the Yellow Pages as Restaurants, but were included in our Takeaway food outlet category.

### 2.3 Spatial unit of analysis

Geocoded addresses were mapped using a geographical information system (GIS), ArcGIS 10 (ESRI Inc., Redlands, CA). Points were overlaid onto 2001 electoral ward boundaries for Norfolk (*n*=205), downloaded from UKBORDERS (http://edina.ac.uk/census). Counts of food outlets by ward were standardised per 10,000 persons using population data from the 2001 UK Census. Wards were divided into tertiles of deprivation using the 2001 Townsend index (1=least deprived, 3=most deprived). The Townsend index is a composite score of area-level material deprivation, calculated from four Census variables: percentage unemployment among economically active adults aged 16 and over; percentage overcrowded households; percentage no car/van ownership; percentage non-home owners (University of Southampton, 2008). Due to changing electoral ward boundaries, we were only able to use the 2001 deprivation estimates, rather than capturing deprivation at multiple time points across the study period. All references to deprivation tertiles are therefore referring to 2001 area deprivation.

### 2.4 Statistical analysis

For analysis of takeaway food outlets, we tested for significant effects of time and of deprivation tertile on mean outlet density per 10,000 persons using a one-way repeated measures analysis of variance (RMANOVA). We report the most conservative Greenhouse–Geisser estimates. To test whether gradients in takeaway food outlet density over time differed significantly between deprivation tertiles, we included an interaction term in our model.

Analysis of supermarket outlets differed because a majority of electoral wards contained no supermarkets. Therefore we coded the presence of any supermarket within each ward into a binary variable (absent/present). We then used a multiple logistic regression model to test the association between supermarket presence, deprivation tertile and time. The analysis was adjusted for ward population and used robust standard errors to control for within-ward clustering.

For both takeaway and supermarket outlets, stability in the number of outlets within wards between 1990 and 2008 was assessed using Spearman׳s rank correlation analysis. As we were interested in identifying any concentration of outlets over time, wards containing no takeaway food outlets (*n*=103) or supermarkets (*n*=174) at either time point were excluded from the correlation analyses. We were also interested in characterising change over time within wards by area deprivation, which was calculated by subtracting the number of takeaway food outlets per ward in 1990 from the number in 2008. All analyses were conducted in Stata 13.0 (StataCorp., 2013).

## 3 Results

### 3.1 Descriptive statistics

Ward populations ranged from 1440 to 10,529 residents (mean (SD) =3886 (2121)) with a total population of 796,728. Townsend index scores ranged from −4.42 (least deprived) to 10.52 (most deprived), which spanned the 5th and 95th percentile of scores for all of England. Of the 205 electoral wards, 85 had at least one takeaway food outlet in 1990, which rose to 88 wards in 2008. Between 1990 and 2008 there was a 45% increase in the number of takeaway food outlets (265–385), with average density per 10,000 residents increasing from 2.6 (SD=4.7) to 3.8 (SD=6.3). In 1990, 26 wards had one or more supermarkets, which increased to 30 wards in 2008. Over the study period, supermarkets increased in number from 31–40, a 29% increase, and average density increased from 0.2 (SD=0.8) to 0.3 (SD=1.0) supermarkets per 10,000 residents.

### 3.2 Changes in takeaway food outlet density by deprivation

**Fig. 1:**
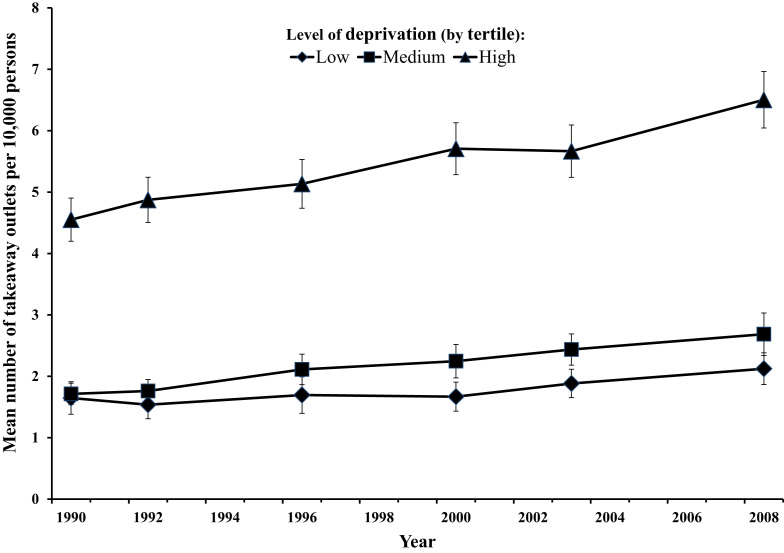
Takeaway food outlet density per 10,000 population by 2001 electoral ward (*n*=205) deprivation tertile, Norfolk, 1990–2008. Data points=mean number of takeaway food outlets per 10,000 population per deprivation tertile; error bars=standard errors.

The most deprived wards had the highest mean density of takeaway food outlets at every time point (Fig. 1). In the modelled results, there was a significant difference in outlet density between deprivation tertiles across the study period (*F*=11.24, *p*<0.001). The most deprived wards saw the largest absolute increase in takeaway food outlet density: in the most deprived tertile density increased by 2 outlets per 10,000 population over the time period from a mean of 4.6 (SD=5.8) in 1990 to 6.5 (SD=7.6) in 2008; a 43% increase. This is in comparison to increases of 1.0 and 0.5 outlets per 10,000 population in the middle and least deprived tertiles, which equate to a 58% and a 30% increase, respectively. These changes in takeaway food outlet density within tertiles over the 18 year period were significant (*F*=12.66, *p*<0.001). The interaction between deprivation tertile and year was non-significant (*F*=1.78, *p*=0.095).

### 3.3 Changes in supermarket density by deprivation

At both baseline and at the final year of the study period, there were a greater number of supermarkets in the most deprived wards than the least deprived. In 1990, five wards in the least deprived and fifteen wards in the most deprived tertiles had at least one supermarket. In 2008, six wards in the least deprived and eighteen wards in the most deprived tertiles had at least one supermarket. However, when we tested supermarket presence by area deprivation across the time period in the adjusted multiple logistic regression model, we found no significant association between deprivation tertile and the odds of supermarket presence (Table 1).

### 3.4 Stability within the food environment over time

There was a strong correlation in the number of takeaway food outlets within wards in 1990 and in 2008 (*r*_*s*_=0.77, *p*<0.001). Wards with relatively high numbers of takeaway food outlets in 1990 tended to have relatively high numbers in 2008, and vice versa for wards containing relatively few. For wards containing supermarkets in either 1990 or 2008 (*n*=31) the Spearman׳s correlation coefficient was non-significant (*r*_*s*_=0.30, *p*=0.11).

**Fig. 2:**
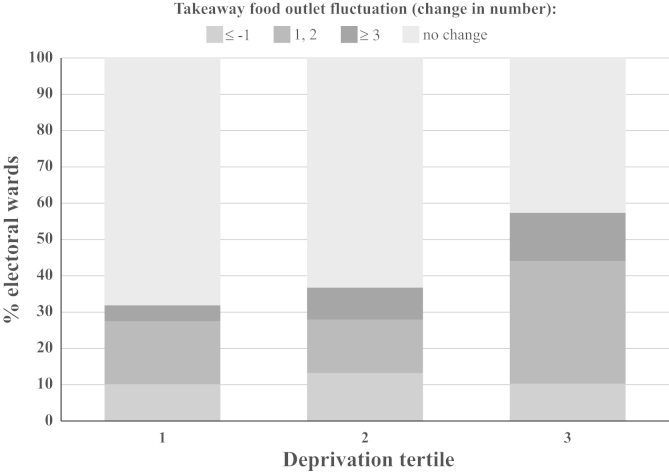
Percentage change in the number of takeaway food outlets between 1990 and 2008 by deprivation tertile, 2001 electoral wards, Norfolk (*n*=205).

Fluctuations in the takeaway food environment within wards were patterned by deprivation, see Fig. 2. The most deprived tertile saw the greatest change overall, with 47% of wards in this tertile gaining takeaway food outlets over this time period, and with a large range in the number of outlets gained or lost (−3 to 9). In comparison, 22% of wards in the least deprived tertile increased their takeaway food outlet numbers over this period, with the extent of ward-level change ranging from −1 to 3.

## 4 Discussion

This study examined two important components of the foodscape (takeaway food outlet and supermarket availability) with respect to area-level deprivation, cross-sectionally and longitudinally in Norfolk, UK. We focused on an 18 year period when meaningful changes in these food retail sectors might have been expected. To our knowledge, this is the first UK study to examine socioeconomic variation in availability of these food outlets over time. Given the increasing scientific and policy focus on the links between food environments, diet and weight (Swinburn et al., 2013; Feng et al., 2010), a more in-depth understanding of deprivation and change in the foodscape was important at this time.

The 29% increase in numbers of supermarkets was consistent with long-term national trends over this period (Clarke et al., 2002; Competition Commission, 2008). The greater number of supermarkets in deprived wards was not statistically significant across the study period. This observation is aligned with previous UK research findings (White et al., 2004), however is in contrast to the majority of the international literature from the United States (Black et al., 2014), New Zealand (Pearce et al., 2008) and Canada (Larsen and Gilliland, 2008; Bertrand et al., 2008). Given the high population provision of large stores and the low absolute numbers of stores in the study area – 40 stores in 2008 – it may not be surprising that supermarket provision is fairly evenly distributed by area deprivation. This said, utilisation of supermarkets will depend on factors other than physical proximity. Perceptions of price, quality and variety have been identified as important determinants of supermarket shopping behaviour in low-income areas (Cummins et al., 2014; Flint et al., 2013), while lower-education and lower-income shoppers are more likely to shop at low-cost supermarkets (Drewnowski et al., 2012).

The positive association of takeaway food outlet density and area deprivation was consistent with the majority of the existing cross-sectional literature (Black et al., 2014; Fleischhacker et al., 2011). Studies from New Zealand (Pearce et al., 2008, 2007), Scotland and England (Macdonald et al., 2007; Cummins et al., 2005) identified greater access to takeaway food outlets in more deprived neighbourhoods. However, a study of out-of-home food outlets in the city of Glasgow, UK, found no difference in density by deprivation (Macintyre et al., 2005). In North America, fast-food restaurant density in high-poverty, non-white urban neighbourhoods was twice that in low-poverty, non-minority urban neighbourhoods (Richardson et al., 2012), with similar findings observed elsewhere in the US (Morland et al., 2002) and Canada (Smoyer-Tomic et al., 2008). In Australia, takeaway food outlet density was 2.5 times greater in the lowest SES areas of Melbourne compared to the wealthiest (Reidpath et al., 2002).

This study moves beyond the existing takeaway food outlet-deprivation literature to examine trends over time. Despite net growth in the overall number of takeaway food outlets across the study area, growth in the number of takeaway food outlets in the most deprived areas was especially strong. Inequalities in takeaway food outlet density were present in 1990 but grew over the study period: density was 2.8 times greater in the most deprived tertile than the least deprived in 1990 and 3.1 times greater in 2008. A comparable study from New Zealand found socioeconomic gradients in densities of all food outlets over forty years to 2005, including supermarkets and takeaway food outlets (Pearce and Day, 2010). A recent longitudinal study from the US found consistently lower access to fast-food restaurants for residents in more deprived neighbourhoods but similar supermarket availability by neighbourhood SES (Richardson et al., 2014). Our findings add to the emerging international literature through an understanding of UK-specific developments in the foodscape.

We found a strong correlation in the number of takeaway food outlets within wards between 1990 and 2008. Furthermore, a higher proportion of deprived wards gained additional outlets and lost fewer over time, relative to the middle and most affluent tertiles. The agglomeration of new takeaway food outlets in neighbourhoods where similar retailers have already located has not been reported in the food environment literature to date. However, the more general phenomenon of takeaway food outlets co-locating in deprived areas has been described previously as a ‘concentration’ effect (Macdonald et al., 2007), while enduring neighbourhood characteristics, particularly with respect to patterns of deprivation, have also been recognised (Sampson, 2012).

### 4.1 Implications for policy

We have noted the persistence of high takeaway food outlet availability in more deprived areas over time, and furthermore, within the same areas. Enduring neighbourhood deprivation, allied with takeaway food outlet provision remaining entrenched along socioeconomic lines, suggests that ‘top down’ intervention may be needed to tackle food environment inequity and to reduce UK diet and health inequalities. A number of English local authorities have implemented or are considering the implementation of policies to regulate the opening of new takeaway food outlets (Ross, 2013). Regulations include planning ordinances to restrict the concentration and clustering of takeaway food outlets, such as limiting adjacency or percentage of store front dedicated to takeaway food on the high street (Greater London Authority, 2012). These initiatives, which so far only apply to new takeaway food outlets, have been endorsed by the Greater London Authority, the National Institute for Health and Care Excellence (NICE), Public Health England and the Academy of Medical Royal Colleges, and so are likely to be widely adopted by other local authorities in future. Internationally, similar regulations have been in place in South Los Angeles since 2008 (Sturm and Cohen, 2009). With regard to supermarkets, our results neither suggested that provision was inequitably distributed nor that additional stores concentrated within the same areas, which limits the policy implications of our findings. Unlike in the US, where supermarket access is more strongly patterned by neighbourhood socio-economic status (Black et al., 2014; Larson et al., 2009), and where policies to improve supermarket provision in low-income areas have gained political traction (Holzman, 2010; The Food Trust, 2013), to our knowledge, there are no current UK-based programmes or policies designed to improve neighbourhood access to supermarkets.

### 4.2 Methodological considerations and limitations

Our study is not without certain limitations. Using telephone directories to collect outlet data may have lacked sensitivity in identifying outlets, and specificity in grouping all takeaway food outlets together when the healthfulness of the food available may vary (Lake et al., 2012). Further, with increased use of the internet as a means of advertising in recent decades, such as the online Yellow Pages (Yell.com), the sensitivity of our method may have decreased over the study period. This said, the accuracy of the Yellow Pages was at least equal to Yell.com in 2010 (Lake et al., 2010), and such changes would have only resulted in an underestimation of food environment change. We were also unable to access Yellow Pages for all years across the study period. While using commercial directories to measure the food environment can present such issues, they have been used for this purpose in a number of other studies (Macdonald et al., 2007; Ball et al., 2009; Burgoine et al., 2009).

Due to changing ward boundaries, we were limited to using ward-based 2001 population and deprivation estimates across all time points studied. We considered that these 2001 estimates, which were central to our study period (1990–2008), would best represent both population and deprivation. However, this approach may have introduced some error into our estimates of outlet density, and so future studies should utilise data where this information has been captured at multiple time points. This said, the spatial patterning of deprivation across Norfolk in 1991 and 2001 do appear visually similar (Supplementary Fig. 1). As mentioned, the 2001 wards in Norfolk spanned the 5th and the 95th percentile of the 2001 Townsend index scores for England, which was also the case for the 1991 ward boundaries and 1991 Townsend index scores, indicating heterogeneity at both time points in levels of deprivation present in Norfolk. Further, it is also reasonable to expect that areas remain relatively socioeconomically similar over time (Sampson and Morenoff, 2006). Our use of administrative boundaries also makes our findings vulnerable to the modifiable areal unit problem. However, our findings are largely in line with previous individual-level studies of exposure to takeaway food outlets by deprivation (Fraser et al., 2010). Our findings may not be generalisable to other regions of the UK. However, the county of Norfolk does have similar characteristics to other regions of the UK and elsewhere, and our findings are aligned with those from similar cross-sectional studies in other settings. Finally, due to our ecological study design, we could not establish causality between area deprivation and the foodscape. While we suggest that neighbourhood socioeconomic status influenced the location of outlets, it may be that less healthful foodscapes attracted more deprived residents; those less concerned with healthy lifestyle behaviours or seeking low-skilled paid employment (Kwate, 2008).

## 5 Conclusions

This study examined change in neighbourhood takeaway food outlet density and supermarket access over time and with respect to 2001 area-level deprivation in the UK. Previous cross-sectional work has prohibited considerations of increased or reduced socioeconomic inequalities in local food environment characteristics over time. Although supermarket presence did not vary significantly by deprivation tertile, significant differences were observed in the density of takeaway food outlets across deprivation tertiles. Deprived areas had the greatest density of takeaway food outlets, and moreover had the greatest increase in takeaway food outlet density across the study period. Our findings support the hypothesis that socioeconomic differences in takeaway food outlet access might partially explain observed socioeconomic differences in diet and body weight. Government planning policy may be required to alter these current and persisting inequities.

## Figures and Tables

**Table 1:** Odds^a^ of supermarket presence by deprivation tertile and time, electoral wards Norfolk 1990–2008, *n*=205.

	**Odds ratio**	**95% CI**	***p***
**Tertile 1 (least deprived)**	**ref**		
**Tertile 2**	**1.26**	**0.48, 3.31**	**0.65**
**Tertile 3 (most deprived)**	**1.87**	**0.69, 5.06**	**0.22**
**Time**^b^	**1.03**	**1.00, 1.05**	**0.03**

a Model adjusted for ward population and for within-ward clustering over time.

b Time as interval between sample years.
