# Supplementary material for: Area deprivation and the food environment over time: A repeated cross-sectional study on takeaway outlet density and supermarket presence in Norfolk, UK, 1990–2008
Source: Health Place. 2015 May;33:142–7. doi: 10.1016/j.healthplace.2015.02.012 (PMC4415115; doi:10.1016/j.healthplace.2015.02.012)
Supplement: Supplementary file 1 — Supplementary Material [file mmc1.pdf]

Maguire, E.R., Burgoine, T. and Monsivais, P. Area deprivation and the food environment over time: A repeated cross-sectional study on takeaway outlet density and supermarket presence in Norfolk, UK, 1990-2008. Health & Place (2015), http://dx.doi.org/10.1016/j.healthplace.2015.02.012

Supplementary Appendix 1: Missing street number geocoding sensitivity analysis.

When food outlet addresses from the Yellow Pages were missing a street number (e.g. Fish Shop, High Street, Norfolk), we attributed street number 1 to these records (e.g. Fish Shop, 1 High Street, Norfolk). This was necessary to allow geocoding using Ordnance Survey's address lookup table for Norfolk. Due to the relatively short street segments typically found in the UK, we assumed that this modelled address would be geographically proximal to the outlet's actual (but unknown) address. Further, as food outlet locations were being attributed to administrative boundaries (electoral wards), some degree of locational error between modelled and actual address would be effectively masked. Lastly, if the locational error between modelled and actual address resulted in the food outlet being allocated into an incorrect ward, our results and conclusions would remain valid and unchanged if the incorrectly attributed ward was of the same level of deprivation. It was however necessary to validate these assumptions.

Using our takeaway food outlet address records from 2000, for which we had actual complete address records (n=317, 95%), and therefore actual attributions to electoral wards, we removed the street numbers and replaced with street number 1 throughout. This effectively created a modelled address dataset that could be compared to the actual address dataset. We used Ordnance Survey's address lookup table to geocode, map and attribute these modelled addresses to 2001 electoral ward boundaries. The results comparing modelled to actual address locations are shown below:

**Modelled vs Actual address location comparison**	**n (%)**
Same ward	230 (72.6)
Different ward	87 (27.4)
Different ward, same deprivation tertile	51 (16.1)
Different ward, different deprivation tertile	36 (11.4)

The majority (73%) of modelled addresses were attributed to the same ward as their actual address equivalent. 16% of modelled addresses were attributed to a different ward, but of the same deprivation tertile as their actual address equivalent. Only 11% of modelled addresses were attributed to a different ward with a different level of deprivation. In our data for 2000, which we consider to be representative of food outlet data in other years, we had incomplete address data for 16 food outlets. This means that 1.8 outlets (assuming 11% locational error rate), or 0.5% of all 333 takeaway food outlets present in Norfolk in 2000, would have been attributed to a ward sufficiently different to the ward of its actual location as to impact upon our results.
