# Supplementary material for: Area deprivation and the food environment over time: A repeated cross-sectional study on takeaway outlet density and supermarket presence in Norfolk, UK, 1990–2008
Source: Health Place. 2015 May;33:142–7. doi: 10.1016/j.healthplace.2015.02.012 (PMC4415115; doi:10.1016/j.healthplace.2015.02.012)

Maguire, E.R., Burgoine, T. and Monsivais, P. Area deprivation and the food environment over time: A repeated cross-sectional study on takeaway outlet density and supermarket presence in Norfolk, UK, 1990-2008. Health & Place (2015), http://dx.doi.org/10.1016/j.healthplace.2015.02.012

Supplementary Figure 1: Thematic maps of Norfolk, UK, 1991 and 2001, quartiles of 2001 Townsend Deprivation Score by electoral ward.

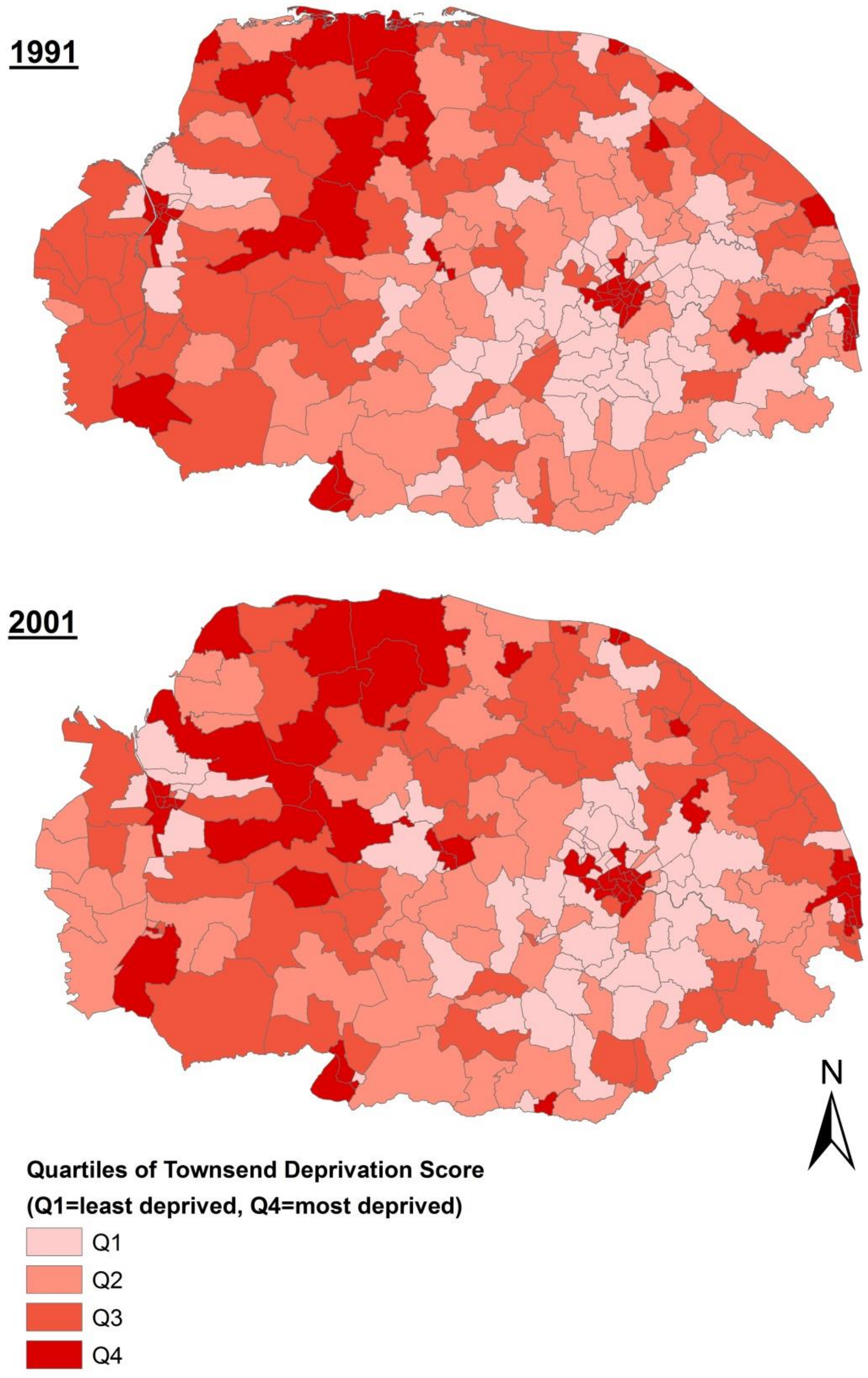

Supplement: Supplementary file 2 — Supplementary Material [file mmc2.pdf]
